# Tandem-biocatalysis reactors constructed by topological evolution of CaCO_3_ particles into hollow metal hydroxide spheres

**DOI:** 10.1038/s41467-023-42649-w

**Published:** 2023-10-26

**Authors:** Sang Yeong Han, Nayoung Kim, Gyeongwon Yun, Hojae Lee, Insung S. Choi

**Affiliations:** 1 Center for Cell-Encapsulation Research, Department of Chemistry, KAIST, Daejeon, 34141 Korea; 2 Department of Chemistry, Hallym University, Chuncheon, 24252 Korea

**Keywords:** Biomaterials, Immobilized enzymes, Biocatalysis

## Abstract

Despite remarkable advances in the design and synthesis of hollow inorganic spheres (HISs), the harsh synthetic conditions have precluded the applications of HISs to biochemical and biological fields. Herein we report a biocompatible strategy for synthesizing metal hydroxide HISs (MH-HISs) by simply mixing CaCO_3_ particles with metal ions in water. The ion-exchange reaction between Ca^2+^ and metal ions leads to the structural and chemical evolution from solid CaCO_3_ particles to hollow MH-HISs via core-shell and yolk-shell structures, while enabling the encapsulation of enzymes to the shells without loss of catalytic activities. The biocompatible protocol makes multienzymatic cascade reactions achievable, with great recyclability due to mechanical durability of MH-HISs.

## Abstract (web summary)

Hollow inorganic spheres (HISs) hold potential in various technological areas including biocatalysis and biomedicine, but the harsh synthetic conditions have precluded the use of HISs in biological fields. Here, the authors report a biocompatible strategy for synthesizing metal hydroxide HISs that can function as tandem-biocatalytic reactors.

## Introduction

Hollow inorganic spheres (HISs), composed of metals, metal oxides, metal sulfides, metal hydroxides, or others, constitute functional materials uniquely featured by large empty spaces inside distinct shells^1–3^. Their intrinsic physical properties, such as low mass density, large surface area, short mass-/charge-transport length, and mechanical stability, make HISs promising candidates in various technological areas, particularly in the area of energy storage, conversion, and production^1,2,4–6^. For example, HISs have intensively been used as electrode materials for the construction of lithium-ion batteries and supercapacitors, in which their commodious interior spaces alleviate the structural deformations that occur during charge and discharge processes, such as destructive volume expansion and contraction, and improve the device’s mechanical and electrochemical stability^7^. Moreover, the thin shells effectively shorten diffusion pathways for both charges and ions, leading to high-rate capacity^8^.

In addition to the intensively exploited, energy-related potential, HISs would advance the bio-related fields, including biocatalysis, biomedicine, and systems biology, were established the methods for biocompatibly confining biochemically functional entities to the HIS’s voids and/or shells. The mechanical durability of inorganic shells would ensure the structural and functional integrity of the biological entities (e.g., enzymes, biotherapeutic agents, and even cells), encapsulated in the porous shells and internal voids, during storage, transport, and use^9–16^. Tandem-biocatalysis reactors could be constructed, based on HISs, by spatially compartmentalizing enzymes and co-factors in the HIS structures^17^. Note that compartmentalization of bioentities in inorganic architectures is rather not a new concept; the iron sulfide (FeS) membrane has been proposed as the first proto-membrane for life, serving as a protective barrier against environmental changes, in natural evolution^18–20^. However, the conventional chemical methods for HIS fabrication^1,2,21–27^―based on template removal, Kirkendall effect, Ostwald ripening, and galvanic replacement―unavoidably require harsh synthetic conditions, such as high temperatures (e.g., for calcination or solvothermal processes) and extreme acidity/basicity (e.g., HF, HCl, or NaOH), in addition to time-consuming, cumbersome operations, so far limiting their applications to abiotic compounds and materials. Recent examples include the fabrication of Fe_2_O_3_-zeolite HISs, catalytically active in gasoline production by the Fischer-Tropsch synthesis, which involves calcination at 550 °C and hydrothermal treatment at 190 °C^28^. As an alternative, metal-organic frameworks (MOFs) have emerged as soft templates and/or shell components for hollow shell formation^29–31^, although the preparation of MOFs still faces issues with harsh reaction conditions and instability in an aqueous solution.

**Fig. 1:**
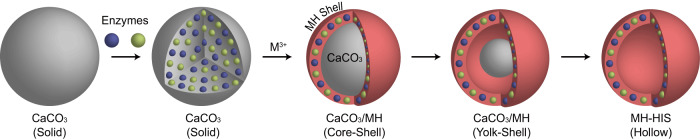
Schematic for topological evolution of CaCO_3_ particles into MH-HISs under physiologically relevant conditions through ion-exchange reactions. Solid CaCO_3_ particles are converted sequentially to CaCO_3_/MH core-shell, CaCO_3_/MH yolk-shell, and MH hollow structures through precisely controlled ion movement and exchange of Ca^2+^ and metal ions (M^3+^) under biocompatible conditions. Enzymes are encapsulated in the shells for multienzymatic cascade reactions.

In this work, we have developed a highly biocompatible method for fabricating metal hydroxide HISs (MH-HISs), which encapsulate multiple enzymes for cascade reactions through efficient substrate channeling. Our strategy is based on the chemical evolution of calcium carbonate (CaCO_3_) particles into MH-HISs under physiologically relevant conditions (Fig. 1). Specifically, we report that solid CaCO_3_ particles gradually transform into CaCO_3_/MH core-shell, CaCO_3_/MH yolk-shell, and MH hollow topologies by controlled exchange of Ca^2+^ and metal ions (e.g., Fe^3+^, Ru^3+^, and V^3+^), serving as biocompatible sacrificial templates for the construction of biocatalytic MH-HISs that contain multiple enzymes in the shells. The open voids of MH-HISs facilitate substrate diffusion, enhancing their potential applications in enzymatic cascade reactions.

## Results

### Formation and characterizations of iron hydroxide HISs (Fe-HISs)

**Fig. 2:**
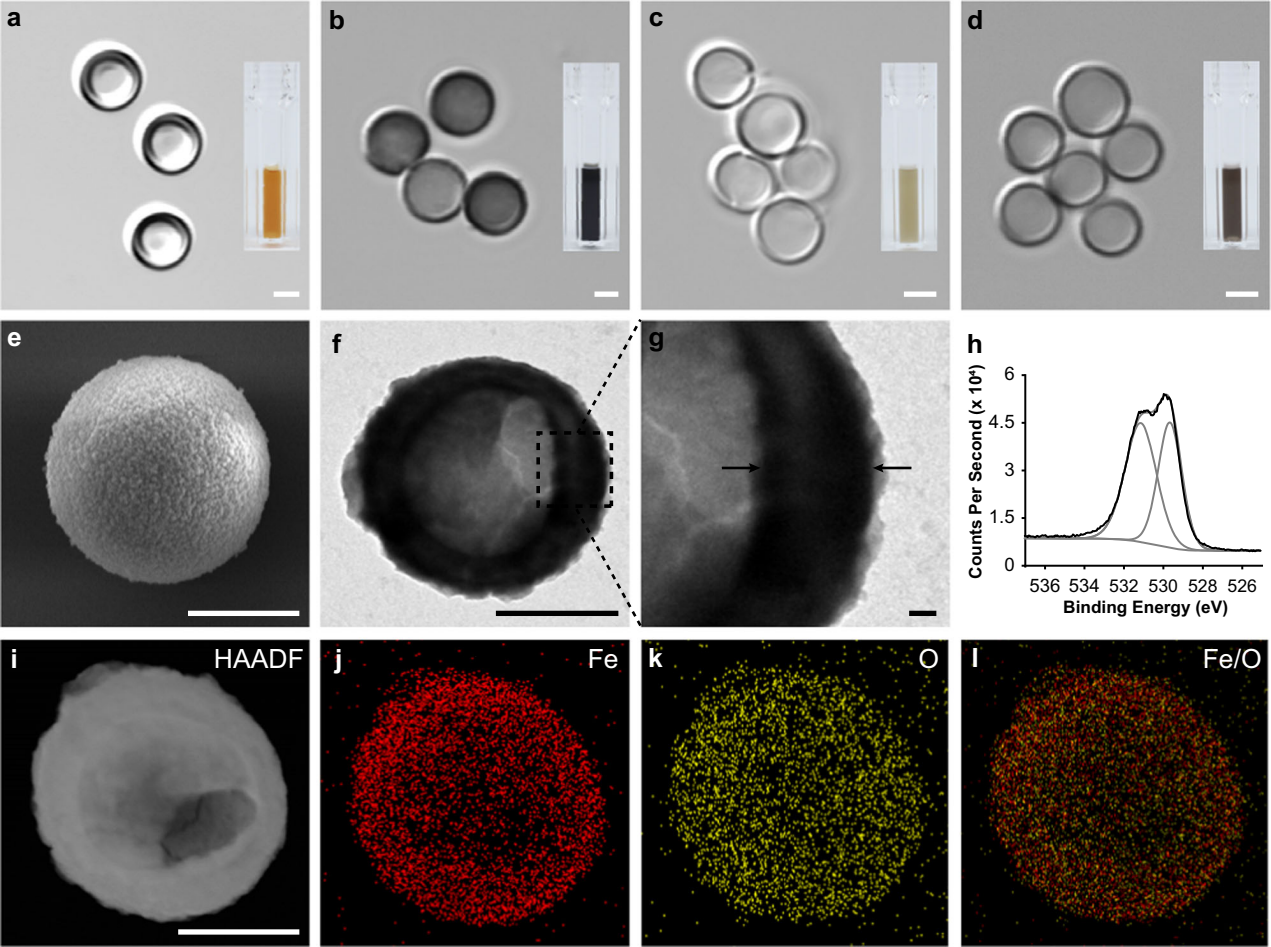
Formation and characterizations of MH-HISs. **a**–**d** DIC images of Fe-HISs, Ru-HISs, V-HISs, and Fe/Ru/V-HISs. Insets: photographs of MH-HIS suspensions. **e**–**l** Characterizations of Fe-HISs. **e**–**g** FE-SEM and TEM images. Black arrows in (**g**) indicate the porous shell. **h** Narrow-scan XPS spectrum showing the presence of Fe–O (529.7 eV) and Fe–OH (531.2 eV) bonds. **i**–**l** High-angle annular dark-field (HAADF) and EDX mapping images. Scale bar: 2 µm (**a**–**d**), 1 µm (**e**, **f**, **i**–**l**), and 100 nm (**g**). The experiments were repeated independently five times with similar results.

We describe the synthesis and characterizations of Fe-HISs as a showcase model (other MH-HISs, such as Ru-, V-, and Fe/Ru/V-HISs, are described in the Methods and Supplementary Information) (Fig. 2 and Supplementary Figs. 1, 2). In short, we delightedly found that Fe-HISs were readily produced, within a short period of time, by simply mixing solid CaCO_3_ particles and FeCl_3_ in deionized (DI) water (concentrations: [CaCO_3_ particle] = 5 mg mL^−1^; [Fe^3+^] = 25 mM). The aqueous suspension of CaCO_3_ particles (diameter: 3-4 µm) turned orange to the naked eyes right after mixing (Fig. 2a, inset). It is noteworthy that the HIS formation was completed within 1 min at ambient temperature.

The successful formation of Fe-HISs was verified by various characterization methods, such as confocal laser-scanning microscopy (CLSM) with differential interference contrast (DIC) mode, field-emission scanning electron microscopy (FE-SEM), and transmission electron microscopy (TEM). Hollow spherical structures, similar to intact CaCO_3_ particles in size, were readily observed in DIC images (Fig. 2a). As characterization examples, the FE-SEM and TEM images of dried Fe-HIS samples supported the hollow nature of the samples (Fig. 2e, f). The shell thickness was determined to be 291 ± 73 nm based on the TEM analysis (Fig. 2g). It is noted that the Fe-HISs are mechanically durable enough to maintain their structural integrity under sample preparation and characterization conditions. Furthermore, the chemical-evolution protocol could be extended to different sizes of CaCO_3_ particles in the formation of Fe-HISs (Supplementary Fig. 3).

Fourier-transform infrared (FT-IR) spectra of Fe-HISs showed signature bands at 1384 (for *v*(Fe–OH)), 1040 (for *v*(Fe–O)), and 473 cm^−1^ (for *v*(Fe–O)) with concomitant disappearance of the bands at 1440 and 880 cm^−1^ assigned to the CO_3_^2-^ vibrations of the CaCO_3_ core (Supplementary Fig. 4)^32,33^. The presence of iron hydroxide species in the HIS was supported by X-ray photoelectron spectroscopic (XPS) analysis, which showed the peaks for Fe–O (at 529.7 eV) and Fe–OH bonds (at 531.2 eV) as well as the peaks for Fe^3+^ (at 711 and 724 eV) (Fig. 2h and Supplementary Fig. 5)^34,35^. The energy-dispersive X-ray (EDX) mapping clearly indicated the uniform distribution of Fe and O over Fe-HISs (Fig. 2i–l). The ζ-potential value was changed to +19.7 mV from −5.3 mV, implying ion exchanges between CaCO_3_ particles and Fe^3+^ (Supplementary Fig. 2). Based on the characterizations, we propose a working reaction for Fe-HIS formation as 2FeCl_3_ + 3CaCO_3_ + 9H_2_O → 2Fe(OH)_3_(H_2_O)_3_ + 3CO_2_ + 3CaCl_2_. The ion-exchange reaction of Ca^2+^ and Fe^3+^ would be thermodynamically favorable, as the solubility product constant (*K*_sp_) of CaCO_3_ (3.3 × 10^−9^) is much higher than that of Fe(OH)_3_ or FeO(OH)•*n*H_2_O (2.5 × 10^–39^)^36,37^. The gaseous phase of the reaction changed an aqueous solution of bromothymol blue (BTB), a CO_2_-sensitive pH indicator, from blue to yellow in color, signifying the CO_2_ generation from the HIS-forming reaction (Supplementary Fig. 6).

### Topological evolution to Fe-HISs

**Fig. 3:**
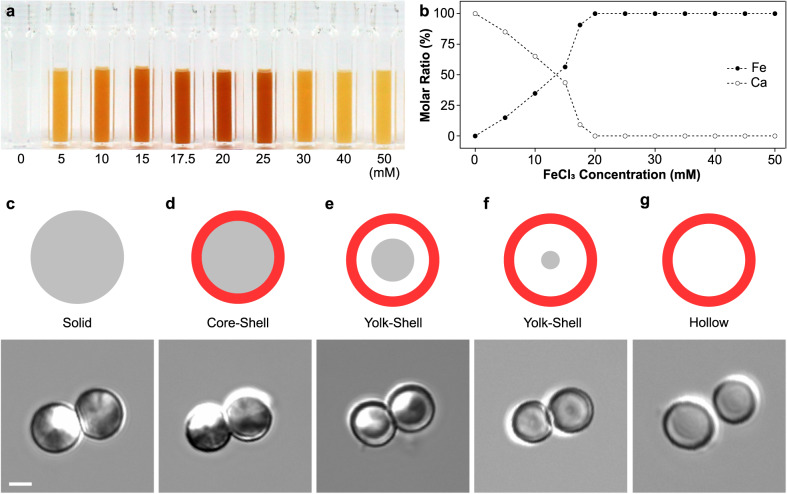
Topological evolution of CaCO_3_ particles into Fe-HISs. **a** Photographs of CaCO_3_-particle suspensions for various FeCl_3_ concentrations. **b** Ion-exchange kinetics for various FeCl_3_ concentrations. **c**–**g** DIC images of resulting products for different FeCl_3_ concentrations (0, 5, 15, 17.5, and 25 mM from left). Scale bar: 2 µm. The experiments were repeated independently five times with similar results.

To investigate the structural evolution from CaCO_3_ particles to Fe-HISs, we varied the FeCl_3_ concentration from 5 to 50 mM. The concentration of CaCO_3_ particles was fixed to be 5 mg mL^−1^. With the increase in the Fe^3+^ concentration, the color of the mixture solution darkened up to 20 mM of FeCl_3_ and then gradually brightened afterwards (Fig. 3a). The photographs were taken 5 min after mixing.

The ion-exchange kinetics was monitored by inductively coupled plasma optical emission spectrometry (ICP-OES), in which the molar fractions of Ca^2+^ (*N*_Ca_: [Ca]/([Ca] + [Fe])) and Fe^3+^ ions (*Ν*_Fe_: [Fe]/([Ca] + [Fe]) in the product sample were calculated with varied FeCl_3_ concentrations (Fig. 3b). The ICP-OES analysis revealed that the Ca^2+^-to-Fe^3+^ substitution rate, inferred from the molar-fraction values, increased linearly for the [Fe^3+^] range of 0 to 20 mM, after which the rate flattened. The reaction time (e.g., 1 min or 180 min) had a negligible effect on *N*_Ca_ and *N*_Fe_ values, confirming that the ion-exchange process was fast.

Considering that the rapid ion movement and exchange would have strong impact on the topological transformation in our system, we investigated the structural evolution, with different Fe^3+^ concentrations, by DIC and EDX mapping analyses (Fig. 3c–g and Supplementary Figs. 7, 8). The analyses clearly showed that the Fe^3+^ concentration critically determined the final structures from core-shell and yolk-shell to HISs. For example, at a low FeCl_3_ concentration (i.e., 5 mM), the ion-exchange reaction between Ca^2+^ and Fe^3+^ occurred predominantly and locally on the surface of CaCO_3_ particles, and the accumulation of Fe^3+^ ions at the particle surfaces led to the formation of CaCO_3_/Fe core-shell particles (Fig. 3d and Supplementary Fig. 8b). In this case, 15% of Ca^2+^ in CaCO_3_ particles was substituted with Fe^3+^ based on the ICP-OES analysis (Fig. 3b). The FE-SEM analysis further confirmed the presence of Fe shells on CaCO_3_ cores (Supplementary Fig. 9). As the FeCl_3_ concentration increased, the ion-exchange reaction progressed inward, initiating the CaCO_3_-core etching. As a representative, at 15 mM of FeCl_3_, 56% of Ca^2+^ was substituted with Fe^3+^, and the inner CaCO_3_ core was observed to be etched uniformly, producing the CaCO_3_/Fe yolk-shell structure (Fig. 3e and Supplementary Fig. 8c). Further increase of the FeCl_3_ concentration gradually reduced the CaCO_3_-core size (e.g., at 17.5 mM of FeCl_3_, 91% Ca^2+^ substitution) (Fig. 3f and Supplementary Fig. 8d), and the CaCO_3_ particle finalized its topological transformation to Fe-HISs with complete core etching (at 25 mM of FeCl_3_, 100% Ca^2+^ substitution) (Fig. 3g and Supplementary Fig. 8e). These results indicated that the observed structural variations were attributed to the difference in reaction rates of shell formation and core etching.

We also followed the topological evolution of CaCO_3_ particles to Fe-HISs at the fixed FeCl_3_ concentration (25 mM) by time-resolved DIC images (Supplementary Fig. 10). The images, taken every 1 sec, fully supported the structural transformation from solid CaCO_3_ particles to core-shell, yolk-shell, and hollow structures in a consecutive manner. It was also found that the reaction was fast, and the topological evolution to Fe-HISs was completed within 4 s. In addition to the chemical-evolution approach to the formation of Fe-HISs, Fe-HISs could be produced from core-shell and yolk-shell particles by removing the CaCO_3_ cores with ethylenediaminetetraacetic acid (EDTA) (Supplementary Figs. 11, 12). Furthermore, the shell thickness of Fe-HISs could be varied with different FeCl_3_ concentrations (Supplementary Fig. 13). For instance, FeCl_3_ concentrations of 5, 15, and 17.5 mM resulted in the formation of Fe-HISs with shell thicknesses of 78.8, 105.1, and 124.8 nm, respectively. No noticeable compression in the void of Fe-HISs was detected in the solution, even for the Fe-HISs produced with a 5-mM Fe^3+^ solution.

### Encapsulation of biological entities in Fe-HISs

Nature has developed complex but orchestrated networks of biological entities that specifically interact with each other to control the metabolism and fate of living cells^38–40^. Inspired by the intricately regulated multienzymatic cascade reactions observed in nature, substantial research efforts have been devoted to the development of artificial tandem-biocatalysis reactors, even with their integration with living cells^10,41^. However, there has been limited exploration with HISs in this research direction due to their hostile fabrication conditions.

We confirmed that our topological-evolution strategy enabled the spatially controlled encapsulation of vulnerable biomolecules (i.e., proteins) in HISs. The encapsulation capacity of our system benefitted, in part, from the result that the ion-exchange reaction between CaCO_3_ particles and Fe^3+^, crucial in the HIS formation, was not affected by the cargos preloaded to the CaCO_3_ particles, not to mention the unique characteristics of the system: rapid reaction under biocompatible conditions (Supplementary Fig. 14). All the fabrication processes were carried out in water at room temperature, ensuring biocompatibility. The pH value of a CaCO_3_-particle suspension was initially 9.75, and was observed to decrease to around 7 after Fe-HIS formation, when exposed to FeCl_3_ solutions with concentrations of 5, 15, and 17.5 mM (Supplementary Table 1). We selected a 5-mM FeCl_3_ solution, resulting in a pH of 7.4 after Fe-HIS formation, for subsequent experiments involving biomolecule encapsulation in Fe-HISs.

While there is methodological potential for spatially controlled encapsulation of biomolecules within voids and shells, as demonstrated with bovine serum albumin (Supplementary Fig. 15), rigorous control experiments revealed that enzymes such as glucose oxidase (GOx) and horseradish peroxidase (HRP), initially trapped within the CaCO_3_ particles, leaked uncontrollably and inconsistently from the void of Fe-HISs, especially during EDTA dissolution step, albeit the average pore size of the Fe-HIS shells was measured to be 2.49 nm by the Brunauer–Emmett–Teller (BET) analysis (Supplementary Fig. 16). Consequently, in this work, we focused on the construction of multienzymatic systems with a particular emphasis on the encapsulation of enzymes within the shells (Supplementary Fig. 17). For instance, the CLSM images of 100 individual Fe-HIS_[HRP-fluorescein]_ captured from various batches consistently displayed fully closed ring structures (Supplementary Fig. 18).

### Tandem-biocatalysis reactions in Fe-HISs

After confirming the controlled encapsulation of proteins within Fe-HIS shells, we investigated multienzymatic tandem-biocatalysis reactions with a set of GOx, HRP, and α-amylase (α-Amy) as a model. In this enzyme set, the cascade reaction in Fe-HISs is initiated by α-Amy, hydrolyzing maltodextrin into shorter chains thereof, maltose, and d-glucose, followed by the subsequent reactions catalyzed by GOx and HRP. In the reactions, the aerobic oxidation of d-glucose by GOx produces hydrogen peroxide (H_2_O_2_) and d-gluconic acid, and the generated H_2_O_2_ acts as an oxidizing co-substrate for HRP (Supplementary Fig. 19). The GOx-HRP cascade reaction was performed with 2,2’-azino-bis(3-ethylbenzothiazoline-6-sulfonic acid) diammonium salt (ABTS) as a substrate for HRP. The catalytic activities were monitored by measuring the UV–vis absorbance at 414 nm, for ABTS radical cations (ABTS^+•^) formed by HRP, every 1 min up to 20 min. Briefly, the enzymes were absorbed into CaCO_3_ particles, and then through the chemical evolution, the enzymes were encapsulated in the shells of Fe-HISs. The concentration of each enzyme used for the encapsulation was set to be 10 U for 5 mg mL^−1^ of CaCO_3_ particles. The DIC images revealed that the enzyme encapsulation had an innocuous effect on the Fe-HIS morphology (Supplementary Fig. 20).

**Fig. 4:**
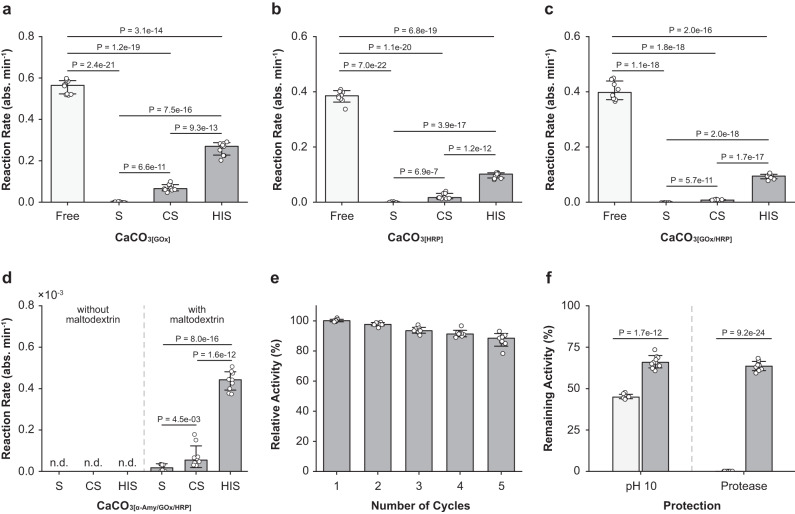
Multienzymatic cascade reactions. **a**–**c** Enzyme-reaction rate of Fe-HIS_[GOx]_, Fe-HIS_[HRP]_, and Fe-HIS_[GOx/HRP]_. **d** Enzyme-reaction rate of Fe-HIS_[α-Amy/GOx/HRP]_ with or without maltodextrin in the ABTS assay solution. Free: free enzymes; S: enzyme-loaded CaCO_3_ solid particles; CS: enzyme-loaded CaCO_3_/Fe core-shell particles; HIS: enzyme-loaded Fe-HISs. n.d.: not detected. **e** Recyclability of Fe-HIS_[GOx/HRP]_. **f** Remaining activity of Fe-HIS_[α-Amy]_ after external assaults (pH 10 and protease). Light gray: free α-Amy; dark gray: Fe-HIS_[α-Amy]_. The statistical data are represented as mean ± SD (unpaired, two-sided Student’s *t* test; *n* = 10 independent experiments).

We first investigated the incorporation efficiency of GOx and catalytic activity of Fe-HIS_[GOx]_ with free HRP (3 U mL^−1^) and ABTS (10 mM) in the media (Fig. 4a and Supplementary Fig. 21). Addition of d-glucose (400 mM) made the Fe-HIS_[GOx]_ suspension rapidly turn dark blue due to the generation of ABTS^+•^, indicating that the catalytic activity of GOx was maintained after the encapsulation process. In the aspect of catalytic activity, 1.39 U of GOx was embedded within the shell, corresponding to 13.9% of the incorporation efficiency. As a control, no color change was detected for Fe-HISs without GOx in the solution of HRP and ABTS. In the case of Fe-HIS_[HRP]_, free GOx (2 U mL^−1^) in the solution was used for the cascade reaction (Fig. 4b), and the analysis indicated that 0.51 U of HRP, corresponding to 5.06% of incorporation efficiency, was encapsulated within the shell in the aspect of activity. The observed catalytic activities corroborated the biocompatible nature of our Fe-HIS system. Notably, the Fe-HIS shell itself exhibited a peroxidase-mimicking catalytic activity, allowing it to efficiently oxidize ABTS to ABTS^+•^, approximately equivalent to about 0.03 U of HRP (Supplementary Fig. 22).

The catalytic activity of Fe-HIS_[GOx]_ was then compared quantitatively with its closed structures, specifically core-shell CaCO_3_/Fe_[GOx]_, to examine the significance of an open interior space for catalytic activity (Fig. 4a). The catalytic reaction was monitored for 20 min to ensure both reaction initiation and saturation. In short, we observed that the GOx activity increased along with the structural evolution. For example, the catalytic activity of GOx increased from 0.38 U for core-shell CaCO_3_/Fe_[GOx]_ to 1.39 U for Fe-HIS_[GOx]_. Additionally, a negative control was implemented by absorbing GOx within CaCO_3_ particles, which exhibited no significant activity. The results clearly showed that the internal void of Fe-HISs could enhance enzyme activities by improving substrate diffusion, even if the enzymes were immobilized in the shell. The enhancement in the catalytic activity was also observed for HRP encapsulation (Fig. 4b): for example, Fe-HIS_[HRP]_ showed 0.51 U of catalytic activity compared with that of 0.12 U within core-shell CaCO_3_/Fe_[HRP]_.

On the other hand, the effects of PSS, Fe^3+^, and EDTA on the catalytic activities were investigated (Supplementary Fig. 23). The catalytic activity of GOx within Fe-HIS_[GOx]_ showed negligible susceptibility to the EDTA treatment. Conversely, the Fe^3+^ treatment elicited a minor reduction in the GOx activity. The addition of PSS to the ABTS solution exhibited no discernible impact on the enzymatic activities. Furthermore, to explore the expandability of our system, Ru^3+^ ions were exploited to synthesize Ru-HISs (Supplementary Fig. 24). Encouragingly, similar to Fe-HISs, Ru-HISs also exhibited a peroxidase-mimicking activity, and Ru-HIS_[GOx]_ could catalyze glucose oxidation and darken the ABTS assay solution.

After confirming the catalytic activities of individual enzyme in the Fe-HIS systems, we studied tandem-biocatalysis reactions of Fe-HISs (Fig. 4c). We concomitantly encapsulated GOx and HRP inside of CaCO_3_ particles and formed Fe-HIS_[GOx/HRP]_, in which both GOx and HRP were distributed in the shells of Fe-HISs (Supplementary Fig. 25). The tandem-biocatalysis reaction of Fe-HIS_[GOx/HRP]_ was observed to be faster than the intermediate structures (CaCO_3[GOx/HRP]_ and CaCO_3_/Fe_[GOx/HRP]_). Specifically, we compared the cascade performances by calculating the reaction rate (i.e., the initial formation rate of ABTS^+•^, *V*: abs. min^−1^) based on the slope during the initial 5 min, as presented in Supplementary Fig. 21 c^42^. The calculation showed that the cascade performance of Fe-HIS_[GOx/HRP]_ was enhanced by 1172%, compared with CaCO_3_/Fe_[GOx/HRP]_ (*V* value: 0.09303 vs. 0.00794), while no meaningful tandem-biocatalysis reaction was observed for CaCO_3[GOx/HRP]_. The versatility of Fe-HISs as a tandem bioreactor was further demonstrated by another multienzymatic cascade reaction, composed of α-Amy, GOx, and HRP (Fig. 4d). A maltodextrin solution (10 mg mL^−1^) was added to a suspension of Fe-HIS_[α-Amy/GOx/HRP]_ containing ABTS. The reaction rate significantly increased along with the structural evolution. In comparison to CaCO_3[α-Amy/GOx/HRP]_, the reaction rate of Fe-HIS_[α-Amy/GOx/HRP]_ was enhanced by 4064%, indicating that the internal void significantly influenced substrate diffusion, with a more pronounced effect with larger substrates. Furthermore, a control experiment without maltodextrin showed no background activity of Fe-HIS_[α-Amy/GOx/HRP]_, confirming the biospecific cascade reaction. The ABTS assay of Fe-HIS_[GOx/HRP]_ with maltodextrin showed no catalytic activity, also indicating the substrate specificity of the reaction (Supplementary Fig. 26). Taken all together, the results clearly confirmed that our synthetic systems constructed a tandem-biocatalysis reactor in a biocompatible fashion, sustaining the catalytic activities for a set of enzymes.

### Recyclability

Enzymes are essential in many industrial processes, such as the synthesis of pharmaceutical, cosmetic, and nutritional compounds, due to their excellent activity, selectivity, and substrate specificity under environmentally friendly conditions (i.e., in DI water)^17,43–45^. However, the difficulty in recovery and reuse, along with structural and functional vulnerability, often impedes industrial applications of enzymes, which would be overcome by the Fe-HIS system. The Fe-HIS-based enzyme encapsulation made our system recyclable with the preservation of enzymatic activities. After a cycle of reaction, Fe-HIS_[GOx/HRP]_ was recovered through centrifugation, and reused for the next cycle of reaction. More than 91.6% of the enzymatic activity of Fe-HIS_[GOx/HRP]_ was retained even after 4 cycles of reactions, with a slight activity decrease to 87.3% after fifth cycle (Fig. 4e). Furthermore, no morphological changes of Fe-HIS_[GOx/HRP]_ were observed after 4 cycles (Supplementary Fig. 27).

We also explored the ability of Fe-HISs to protect the encapsulated enzymes from external stressors, such as protease and pH fluctuation (Fig. 4f). Protease is one of the proteolytic enzymes that can inactivate α-Amy. After 3-h protease treatment to a suspension of Fe-HIS_[α-Amy]_, the reaction was commenced by adding a maltodextrin solution containing GOx, HRP, and ABTS. The catalytic activity of α-Amy in Fe-HIS_[α-Amy]_ decreased to 63.6%, whereas only 0.2% of the activity of free α-Amy was recorded after protease treatment, clearly confirming that the enzymes were protected by Fe-HISs. In addition, the relative catalytic activity of α-Amy in Fe-HIS_[α-Amy]_ was 66.2% after 3-h incubation in a pH-10 sodium carbonate buffer solution, compared with 45.2% for free α-Amy. These observations clearly demonstrated that Fe-HISs could effectively protect the encapsulated enzymes from external stressors.

## Discussion

This work adds a hitherto unreachable or uncharted territory―creation of tandem-biocatalytic reactors―to the repertoire of hollow inorganic spheres (HISs). Uniqueness of our strategy is the controlled structural transformation of solid CaCO_3_ particles to Fe-HISs by precisely designed ion exchange of Ca^2+^ with Fe^3+^ under physiologically relevant conditions. Its biocompatible processes, with rapidity (<1 min), realize the spatially controlled encapsulation of a set of enzymes in the shells, while sustaining catalytic activities. Our synthetic protocol has several advantages for the construction of tandem-biocatalysis reactors: The fabrication process is straightforward and fast, which would be applied seamlessly to diverse bio-related sectors; The vast voids and porous shells would provide sufficient space for the encapsulated biological entities to reorient and diffuse in their lower surface energy states; HISs facilitate the short and efficient diffusion of reactants and reaction intermediates (e.g., gasses and nutrients), enabling the efficient operation of multienzymatic cascade reactions; The mechanical durability guarantees the structural and functional integrity of the encapsulated bioentities, providing exceptional efficiency and recycling for biocatalysis reactions. Furthermore, our system is flexible in compositions, accommodating various metal precursors for HIS construction and entities for encapsulation, widening its applicability to a wide range of areas including biocatalysis, biodiesel production, and bioremediation.

## Methods

### Synthesis of calcium carbonate (CaCO_3_) particles

CaCO_3_ particles were prepared by a rapid precipitation reaction between sodium carbonate (Na_2_CO_3_) and calcium chloride (CaCl_2_). In brief, aqueous solutions of Na_2_CO_3_ (48 µL, 1 M) and poly(sodium 4-styrene sulfonate) (PSS, 4 mL, 2 mg mL^−1^) were mixed in a beaker under ambient conditions. An aqueous CaCl_2_ solution (96 µL, 1 M) was then added quickly to the Na_2_CO_3_-PSS solution under constant stirring, and the mixture was stirred further for 45 s. The resulting CaCO_3_ particles were incubated for 3-10 min to achieve the desired size (3-6 μm). The particle growth was monitored by optical microscopy. The CaCO_3_ particles were washed with DI water three times to remove excess Na_2_CO_3_, PSS, and CaCl_2_. In the washing step, the particles were separated by centrifugation at 2000 *g* for 30 s (Centrifuge 5418, Eppendorf), and the supernatant was removed. The generated CaCO_3_ particles (i.e., PSS-stabilized CaCO_3_ particles) were suspended in 1 mL of DI water. For the formation of calcined CaCO_3_ particles (without PSS), PSS was removed by calcination at 450 °C for 2 h, and the particle powder was stored in a dry solid state. The calcined CaCO_3_ particles were used for FT-IR analysis.

### Fabrication of metal hydroxide-hollow inorganic spheres (MH-HISs)

PSS-stabilized CaCO_3_ particles or calcined CaCO_3_ particles (5 mg) were added to 1 mL of a fresh metal precursor solution (25 mM of FeCl_3_, RuCl_3_, or VCl_3_ in DI water) under ambient conditions. The RuCl_3_ solution was used after filtering with a syringe filter (0.22 μm pore size). For the preparation of Fe/Ru/V-HISs, 1 mL of the multiple-metal precursor solution (final concentration: [Fe^3+^, Ru^3+^, or V^3+^] = 8.333 mM each) was used as a metal source. The suspension was strongly mixed with a vortex mixer for 1 min, and the obtained MH-HISs were washed with DI water three times (2000 *g*, 30 s). MH-HISs were dispersed in DI water (1 mL) and used for CLSM, FE-SEM, and TEM measurements.

### Topological evolution of CaCO_3_ particles to Fe-HISs

CaCO_3_ particles (5 mg) were added to 1 mL of the aqueous FeCl_3_ solution (0, 5, 10, 15, 17.5, 20, 25, 30, 35, 40, 45, or 50 mM) under ambient conditions. After vigorously mixing with a vortex mixer for 1 min, the resulting products were washed with DI water three times (2000 *g*, 30 s) to remove unreacted FeCl_3_. The remaining pellets were dispersed in DI water and used for further characterizations. In the in-situ imaging, an equal volume of the metal precursor solution (50 mM, in DI water) and CaCO_3_ particle suspension (10 mg mL^−1^, in DI water) was sequentially introduced into a confocal dish. The topological transformation of CaCO_3_ particles was followed in real-time, right after mixing, by CLSM.

### Enzyme encapsulation

(1) *Synthesis of enzyme-absorbed CaCO*_*3*_ *particles (CaCO*_*3[Enz]*_ *particles):* PSS-stabilized CaCO_3_ particles were prepared by the aforementioned procedures. Enzymes (GOx-rhodamine or HRP-fluorescein: 1 mg; GOx or HRP: 10 U) were added to 5 mg mL^−1^ of a CaCO_3_ particle suspension, and the mixture was incubated in a tube rotator (30 rpm) for 6 h. For multienzyme encapsulation, GOx, HRP, and α-Amy (10 U each) were added to 5 mg mL^−1^ of a CaCO_3_ particle suspension. After supernatant removal by centrifugation at 2000 *g* for 30 s, the resulting particles (CaCO_3[Enz]_) were used, after washing with DI water three times, for the Fe-HIS formation. (2) *Synthesis of enzyme-encapsulated Fe-HISs:* CaCO_3[Enz]_ particles were added to 1 mL of a FeCl_3_ solution (5 mM, in DI water). The suspension was vigorously mixed with a vortex mixer for 1 min, and the obtained particles (enzyme-loaded CaCO_3_/Fe core-shell particles) were washed with DI water three times (2000 *g*, 30 s). To obtain enzyme-encapsulated Fe-HISs (Fe-HIS_[Enz]_), the core-shell CaCO_3_/Fe_[Enz]_ particles were washed with EDTA (0.1 M, pH 8) and DI water three times each, and the resulting Fe-HIS_[Enz]_ particles were suspended in 1 mL of DI water. Ru-HIS_[GOx]_ was synthesized in the same manner as the synthesis of Fe-HIS_[GOx]_, except that a RuCl_3_ solution (5 mM) was used. (3) *Enzyme-loading efficiency:* The amount of encapsulated GOx or HRP in CaCO_3_ particles was evaluated by measuring UV–vis absorbance. The 1 mg of GOx-rhodamine or HRP-fluorescein was added to 5 mg mL^−1^ of a CaCO_3_ particle suspension, and the mixture solution was incubated for 6 h. The resulting CaCO_3[Enz]_ particles were washed with DI water three times and dissolved by EDTA (0.1 M, pH 8), and the UV–vis absorbance of the dissolved solution at 550 nm or 460 nm was measured. The enzyme-loading efficiency was calculated compared with the absorbance of 1 mg mL^−1^ of the fluorescently-tagged-enzyme solution.

### Tandem-biocatalysis reactions in Fe-HISs

Kinetics assay was performed by the colorimetric ABTS assay in DI water at ambient temperature. The assay solution was prepared by mixing an ABTS solution (0.1 mL, 10 mM, in DI water) and a d-glucose solution (0.1 mL, 400 mM, in DI water) in 0.7 mL of DI water, and added to a 0.1-mL suspension of Fe-HIS_[GOx]_ with free HRP (3 U), Fe-HIS_[HRP]_ with free GOx (2 U), or Fe-HIS_[GOx/HRP]_. All the samples used for the kinetics assay were made based on 5 mg of CaCO_3_ particles in 1 mL of DI water: CaCO_3[Enz]_, core-shell CaCO_3_/Fe_[Enz]_, and Fe-HIS_[Enz]_ were prepared by the aforementioned procedures with 5 mg of CaCO_3_ particles. The enzyme set of free GOx (3 U for Fe-HIS_[GOx]_) or HRP (2 U for Fe-HIS_[HRP]_) was used as a control. The amount of encapsulated GOx or HRP in Fe-HISs was evaluated by using the kinetics assay. For three-enzyme cascade reactions, 0.6 mL of DI water were added 0.1 mL of an Fe-HIS_[α-Amy/GOx/HRP]_ suspension, 0.1 mL of an ABTS solution (10 mM, in DI water), and 0.2 mL of a maltodextrin solution (50 mg mL^−1^, in DI water). The absorbance of the reaction solution was measured at 414 nm every 15 min after removal of the enzyme-loaded Fe-HISs (2000 *g*, 30 s). As controls, intact CaCO_3_ particles, CaCO_3[Enz]_ particles, and core-shell CaCO_3_/Fe_[Enz]_ particles were used.

### Recyclability and protectability studies

For the recyclability study, an Fe-HIS_[GOx/HRP]_ solution was used for tandem-biocatalysis reactions. After 30 min of reaction, Fe-HIS_[GOx/HRP]_ was recovered by centrifugation (2000 *g*, 30 s), washed with DI water, and placed in a fresh substrate solution. The absorbance of the reaction solution was measured at 414 nm after the removal of Fe-HIS_[GOx/HRP]_ (2000 *g*, 30 s). For the protection experiment, Fe-HIS_[α-Amy]_ was exposed to 1 mL of a protease solution (10 mg mL^−1^) or a sodium carbonate solution (pH 10) for 3 h under a tube rotator (30 rpm). After washing with DI water three times, 0.1 mL of the Fe-HIS_[α-Amy]_ suspension was added to 0.9 mL of DI water that contained ABTS (1 mM), free GOx (1 U), free HRP (1 U), and maltodextrin (10 mg mL^−1^). After 3 h of reaction, the absorbance of the reaction solution was measured at 414 nm after removal of Fe-HIS_[α-Amy]_ (2000 *g*, 30 s).

### Statistical analysis

Ten independent samples were prepared, with three distinct measurements conducted for each sample by extracting portions. Data are presented as the mean of the means ± SD, and represented as white circles to denote the mean from each independent experiment. Statistical significance was determined using an unpaired, two-sided Student’s *t*-test (*n* = 10). IBM SPSS Statistics 26 and Origin 2019 were used to perform data analysis and graph generation.

### Reporting summary

Further information on research design is available in the Nature Portfolio Reporting Summary linked to this article.

### Supplementary information

Supplementary Information

Reporting Summary

## Data Availability

The data supporting the findings from this study are available within the article file and its supplementary information.
