# Supplementary material for: Tandem-biocatalysis reactors constructed by topological evolution of CaCO3 particles into hollow metal hydroxide spheres
Source: Nat Commun. 2023 Oct 26;14:6828. doi: 10.1038/s41467-023-42649-w (PMC10603116; doi:10.1038/s41467-023-42649-w)
Supplement: Supplementary file 1 — Supplementary Information [file 41467_2023_42649_MOESM1_ESM.pdf]

Supplementary Information

Tandem-Biocatalysis Reactors Constructed by Topological Evolution of $CaCO_3$ Particles into Hollow Metal Hydroxide Spheres

Sang Yeong Han[1], Nayoung Kim[1], Gyeongwon Yun[1], Hojae Lee[2], and Insung S. Choi[1]*

[1] Center for Cell-Encapsulation Research, Department of Chemistry, KAIST, Daejeon 34141, Korea
[2] Department of Chemistry, Hallym University, Chuncheon 24252, Korea

CONTENTS

- **Experimental Section**
- **Supplementary Table 1:** The pH values of the reactions with different Fe^{3+} concentrations.
- **Supplementary Fig. 1:** FE-SEM images of Ru-HIS, V-HIS, and Fe/Ru/V-HIS.
- **Supplementary Fig. 2:** ζ-potential values of MH-HISs in DI water.
- **Supplementary Fig. 3:** DIC images after Fe-HIS formation with $CaCO_3$ particles of different diameters.
- **Supplementary Fig. 4:** FT-IR spectra of Fe-HISs.
- **Supplementary Fig. 5:** XPS spectrum of Fe-HISs.
- **Supplementary Fig. 6:** Color changes of the BTB solution.
- **Supplementary Fig. 7:** DIC images of calcined $CaCO_3$ particles at different $FeCl_3$ concentrations.
- **Supplementary Fig. 8:** EDX mapping and TEM images of $CaCO_3$ particles at different $FeCl_3$ concentrations.
- **Supplementary Fig. 9:** FE-SEM images of $CaCO_3$ particles before and after $FeCl_3$ (5 mM) treatment.
- **Supplementary Fig. 10:** Time-resolved DIC images.
- **Supplementary Fig. 11:** DIC images of $CaCO_3$ particles, $CaCO_3$/Fe particles, and Fe-HISs before and after EDTA treatment.
- **Supplementary Fig. 12:** FE-SEM, TEM, and EDX-mapping images of Fe-HISs synthesized from $CaCO_3$/Fe core-shell particles ([Fe^{3+}]: 5mM) by EDTA treatment.
- **Supplementary Fig. 13:** AFM and TEM images of Fe-HISs.
- **Supplementary Fig. 14:** BSA encapsulation in the shell of Fe-HISs.
- **Supplementary Fig. 15:** CLSM images of an Fe-HIS encapsulating BSA-fluorescein and BSA-rhodamine.
- **Supplementary Fig. 16:** Nitrogen adsorption-desorption isotherms of $CaCO_3$ particles, $CaCO_3$/Fe particles, and Fe-HISs, and BET surface areas and pore sizes.
- **Supplementary Fig. 17:** Encapsulation of enzymes in the shell of Fe-HISs.
- **Supplementary Fig. 18:** CLSM images of 100 individual Fe-$HIS_{[HRP\text{-}fluorescein]}$ from various batches.
- **Supplementary Fig. 19:** Schematic for a cascade reaction catalyzed by α-Amy, GOx, and HRP.
- **Supplementary Fig. 20:** DIC images of Fe-$HIS_{[GOx]}$, Fe-$HIS_{[HRP]}$, and Fe-$HIS_{[GOx/HRP]}$.
- **Supplementary Fig. 21:** Time-lapse UV-vis absorbance of Fe-$HIS_{[GOx]}$, Fe-$HIS_{[HRP]}$, and Fe-$HIS_{[GOx/HRP]}$ at 414 nm.
- **Supplementary Fig. 22:** Peroxidase-mimicking catalytic activity of Fe-HISs compared with free HRP.
- **Supplementary Fig. 23:** Effect of PSS, Fe^{3+} ion, and EDTA on the activity of GOx within the shell of Fe-HISs.
- **Supplementary Fig. 24:** Time-lapse UV-vis absorbance of Ru-$HIS_{[GOx]}$ and Ru-HIS at 414 nm.
- **Supplementary Fig. 25:** CLSM images of Fe-$HIS_{[GOx\text{-}rhodamine/HRP\text{-}fluorescein]}$.
- **Supplementary Fig. 26:** Time-lapse UV-vis absorbance at 414 nm of the ABTS assay solution containing Fe-$HIS_{[GOx/HRP]}$ with maltodextrin.

- **Supplementary Fig. 27:** TEM images of Fe-$HIS_{[GOx/HRP]}$ before and after five recycling experiments.

Experimental Section

Materials. Calcium chloride dihydrate ($CaCl_2 \cdot 2H_2O$, Sigma-Aldrich), sodium carbonate (Na_2CO_3, ≥99.5%, Sigma-Aldrich), poly(sodium 4-styrene sulfonate) (PSS, average Mw ~70 kDa, Sigma-Aldrich), iron (III) chloride hexahydrate ($FeCl_3 \cdot 6H_2O$, Sigma-Aldrich), ruthenium(III) chloride hydrate ($RuCl_3 \cdot xH_2O$, Sigma-Aldrich), vanadium(III) chloride (VCl_3, 97%, Sigma-Aldrich), sodium hydroxide (NaOH, ≥99.5%, Sigma-Aldrich), bovine serum albumin (BSA, Sigma-Aldrich), NHS-rhodamine (5/6-carboxy-tetramethyl-rhodamine succinimidyl ester, mixed isomer, Thermo Scientific), NHS-fluorescein (5/6-carboxyfluorescein succinimidyl ester, mixed isomer, Thermo Scientific), ethylenediaminetetraacetic acid disodium salt (EDTA, Junsei), phosphate-buffered saline (PBS, 10 mM, pH 7.4, Welgene), a buffer solution, pH 10 (Samchun Chemicals), bromothymol blue (BTB, Sigma-Aldrich), glucose oxidase (GOx, from *Aspergillus niger*, Sigma-Aldrich), horseradish peroxidase (HRP, from *Armoracia rusticana*, Sigma-Aldrich), α-amylase (α-Amy, from *Aspergilus oryzae*, Sigma-Aldrich), D-(+)-glucose (≥99.5%, Sigma-Aldrich), protease (from *Streptomyces griseus*, Sigma-Aldrich), maltodextrin (dextrose equivalent 16.5-19.5, Sigma-Aldrich), 2,2'-azino-bis(3-ethylbenzothiazoline-6-sulfonic acid) diammonium salt (ABTS, ≥98.0%, Sigma-Aldrich), formvar/carbon supported copper grids (200 mesh, Electron Microscopy Sciences), and lacey/carbon copper grids (200 mesh, Electron Microscopy Sciences) were used as received. Gold substrates were prepared by thermal deposition of Ti (5 nm) and Au (100 nm) onto silicon wafers (Sehyoung Wafertech). Deionized (DI) water (18.3 MΩ·cm) from Milli-Q Direct 8 (Millipore) was used.

Characterizations. Confocal laser-scanning microscopy (CLSM) imaging was performed with LSM 700 and LSM 800 (Carl Zeiss). Transmission electron microscopy (TEM) imaging was performed with JEM-2100F (JEOL) and Tecnai F30 (FEI) with an accelerating voltage of 200 and 300 kV. TEM-assisted energy-dispersive X-ray spectroscopy (EDX) mapping analyses were performed with an X-MAX^N 80 (Oxford Instruments) with an accelerating voltage of 200 kV. Field-emission scanning electron microscopy (FE-SEM) imaging was performed with an FEI Inspect F50 microscope (FEI) with an accelerating voltage of 10 kV. In TEM and FE-SEM experiments, MH-HIS suspensions (5 μL) were allowed to be air-dried on formvar/carbon-supported copper grids, lacey/carbon copper grids, and Piranha-cleaned gold plates, respectively. Zeta (ζ)-potentials of MH-HISs were recorded with a Zetasizer Nano ZS (Malvern). Fourier-transform infrared (FT-IR) spectra were obtained with a Nicolet Nexus FTIR spectrophotometer (Thermo Fisher). X-ray photoelectron spectroscopy (XPS) spectra were taken with a Sigma Probe (Thermo VG Scientific). Kinetics of enzymatic reactions was studied with a microplate reader, SpectraMax iD5 (Molecular Devices).

Supplementary Table 1: The pH values of the reactions with different Fe^{3+} concentrations.

**Fe^{3+} concentration**	**After Fe-HIS formation**
5 mM	7.40 ± 0.2
15 mM	6.89 ± 0.1
17.5 mM	6.69 ± 0.2
25 mM	2.92 ± 0.1

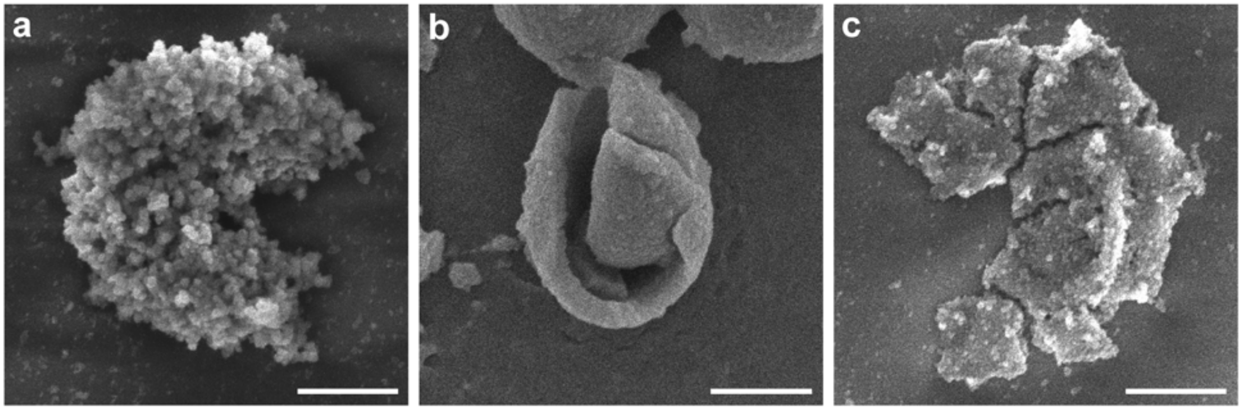

Supplementary Fig. 1: FE-SEM images of (a) Ru-HIS, (b) V-HIS, and (c) Fe/Ru/V-HIS. Scale bar: 2 µm.

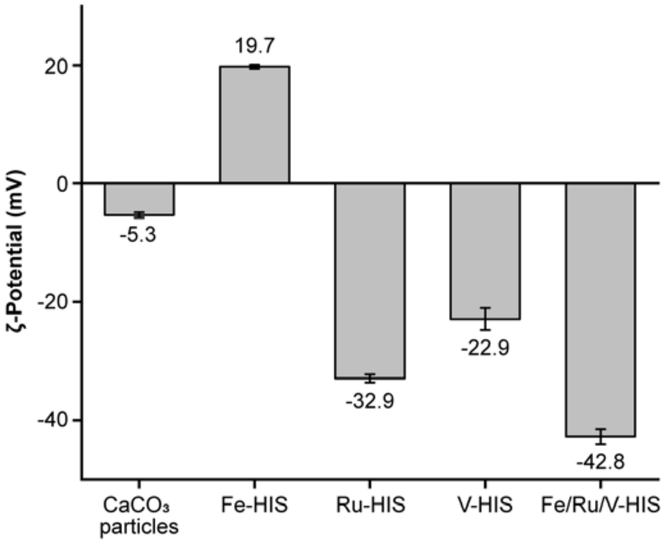

Supplementary Fig. 2: ζ-potential values of intact PSS-stabilized $CaCO_3$ particles, Fe-HISs, Ru-HISs, V-HISs, and Fe/Ru/V-HISs in DI water. Data are represented as mean ± SD.

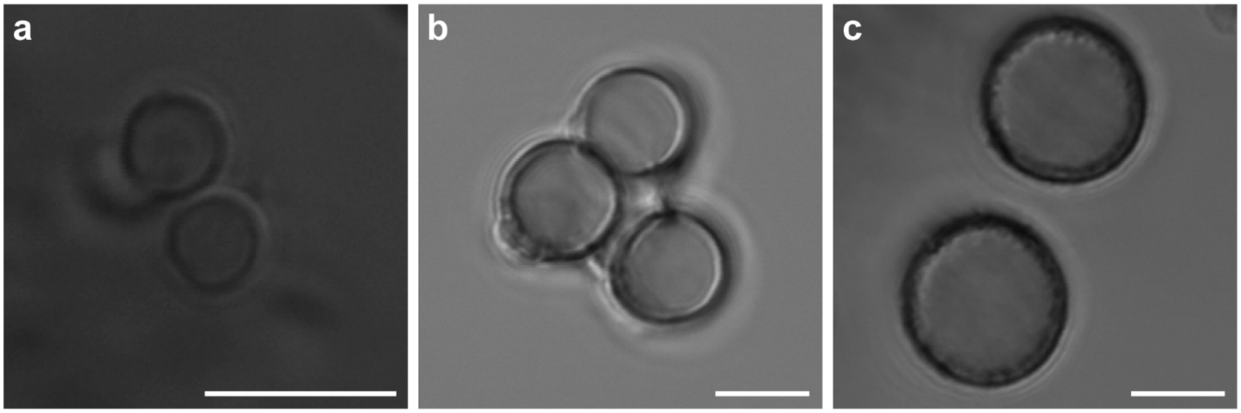

Supplementary Fig. 3: DIC images after Fe-HIS formation with $CaCO_3$ particles of different diameters: (a) 0.5 μm, (b) 5 μm, and (c) 7-8 μm. Scale bar: 5 μm.

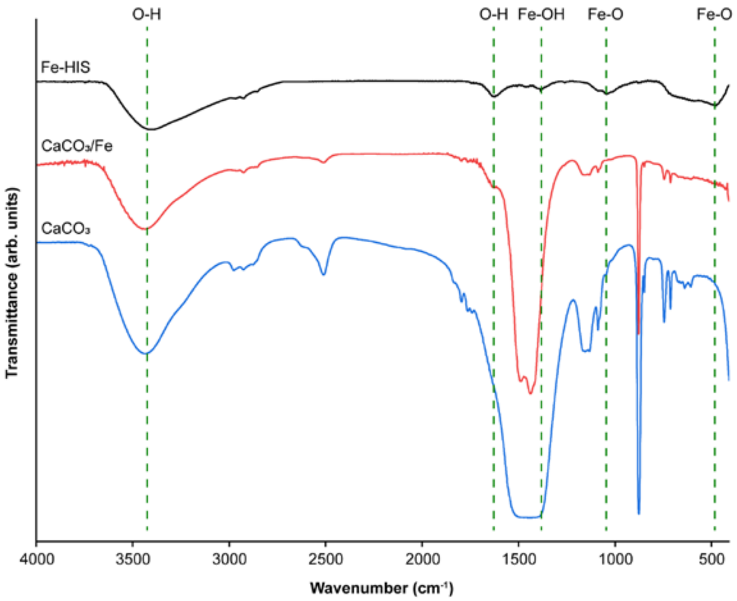

Supplementary Fig. 4: FT-IR spectra of calcined $CaCO_3$ particles, $CaCO_3$/Fe particles, and Fe-HISs.

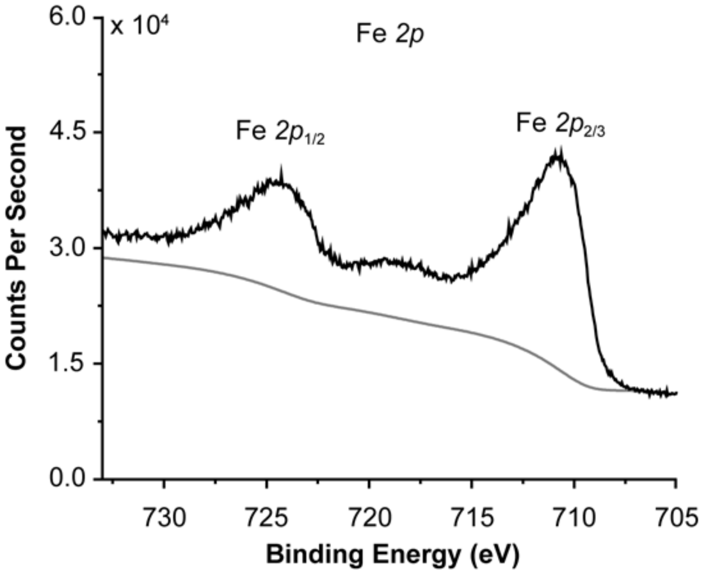

Supplementary Fig. 5: XPS spectrum of Fe-HISs showing the presence of Fe^{3+} at 711 and 724 eV.

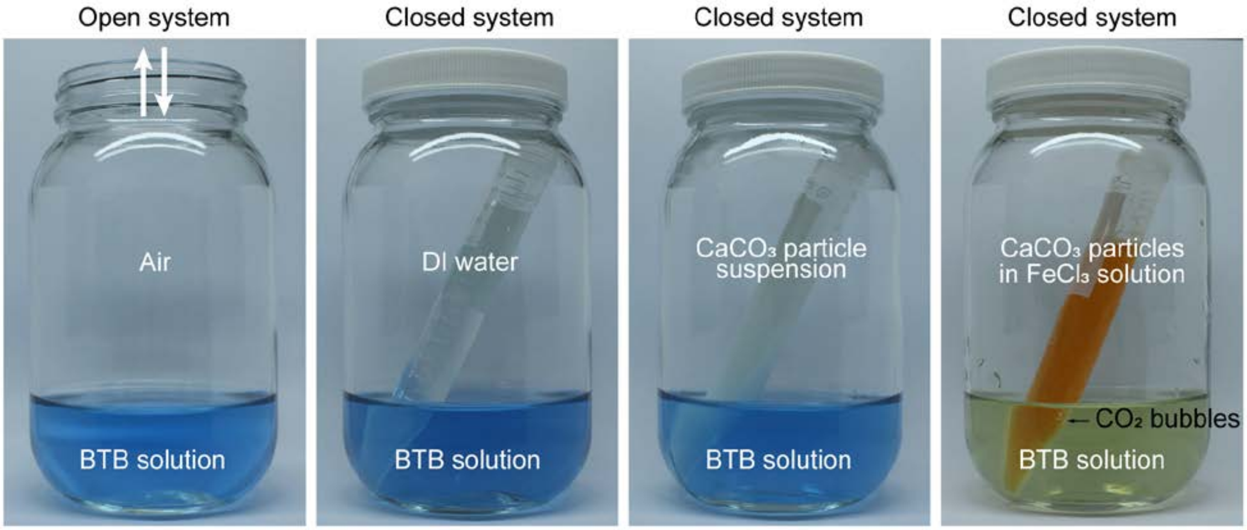

Supplementary Fig. 6: Color changes of the BTB solution by the gaseous phase of the $FeCl_3$-$CaCO_3$ particle solution.

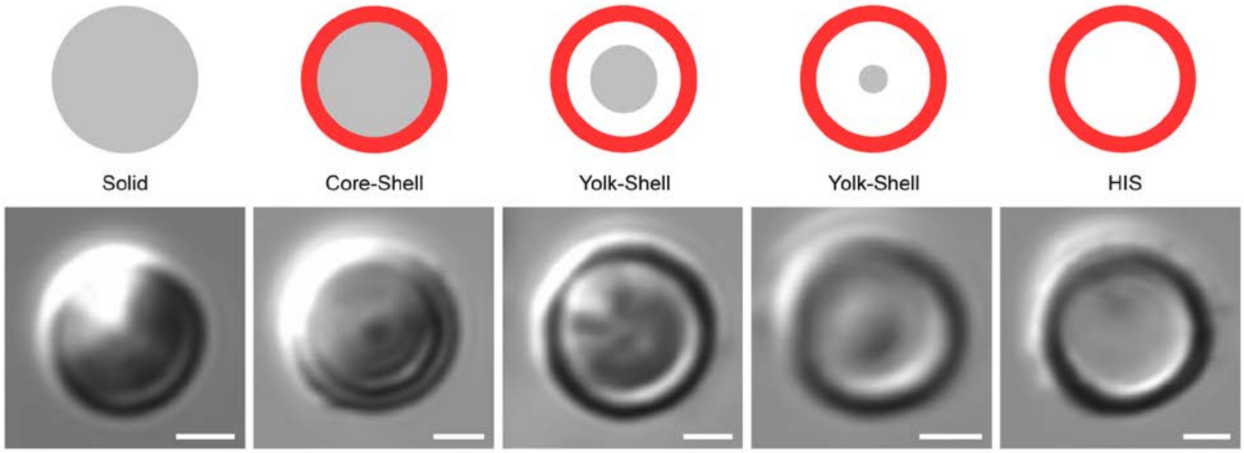

Supplementary Fig. 7: DIC images of calcined $CaCO_3$ particles at different $FeCl_3$ concentrations (from left to right: 0, 5, 15, 17.5, and 25 mM). Scale bar: 1 µm.

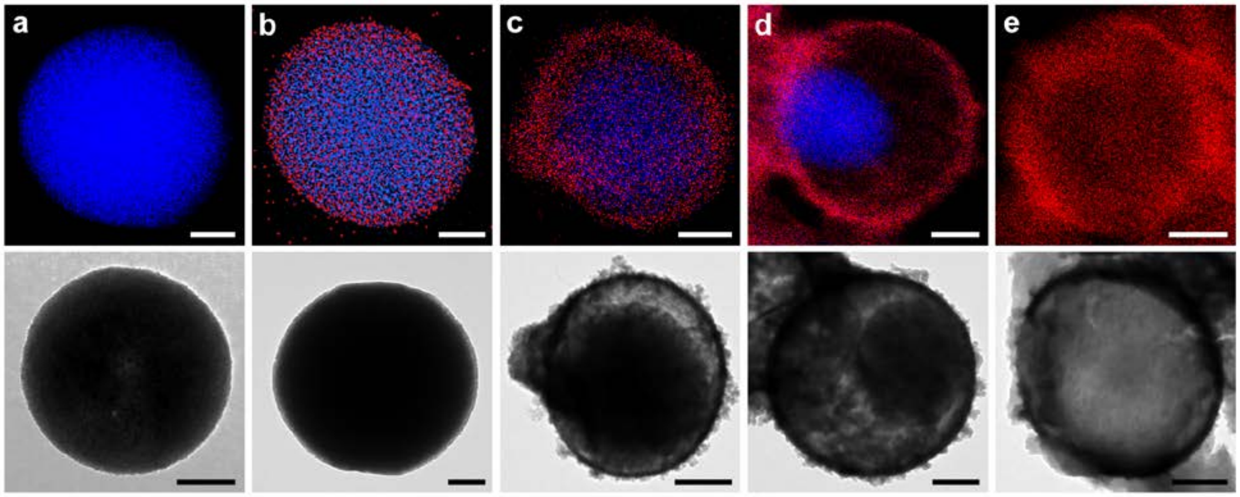

Supplementary Fig. 8: EDX mapping and TEM images of $CaCO_3$ particles at different $FeCl_3$ concentrations (from left to right: 0, 5, 15, 17.5, and 25 mM). Blue: $CaCO_3$; red: Fe shell. Scale bar: 0.5 µm.

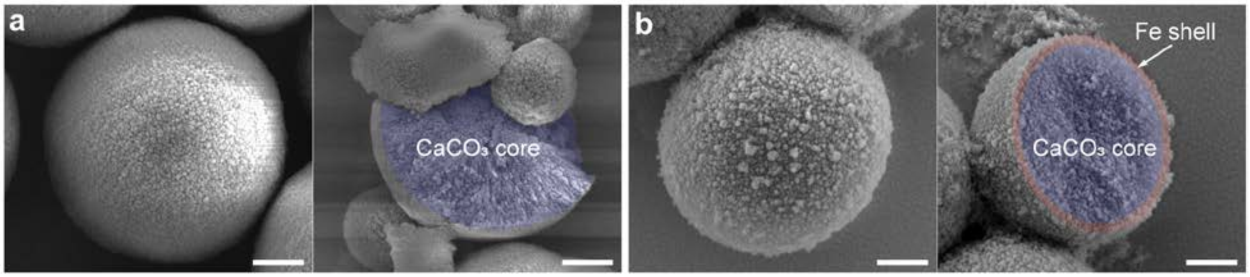

Supplementary Fig. 9: FE-SEM images of $CaCO_3$ particles (a) before and (b) after treatment of $FeCl_3$ (5 mM). Scale bar: 0.5 µm.

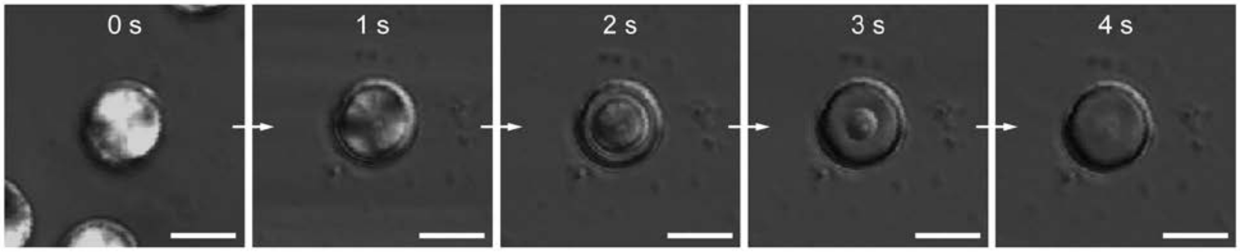

Supplementary Fig. 10: Time-resolved DIC images showing the continuous and sequential transformation of a $CaCO_3$ particle into a Fe-HIS in $FeCl_3$ solution (25 mM). Scale bar: 5 µm.

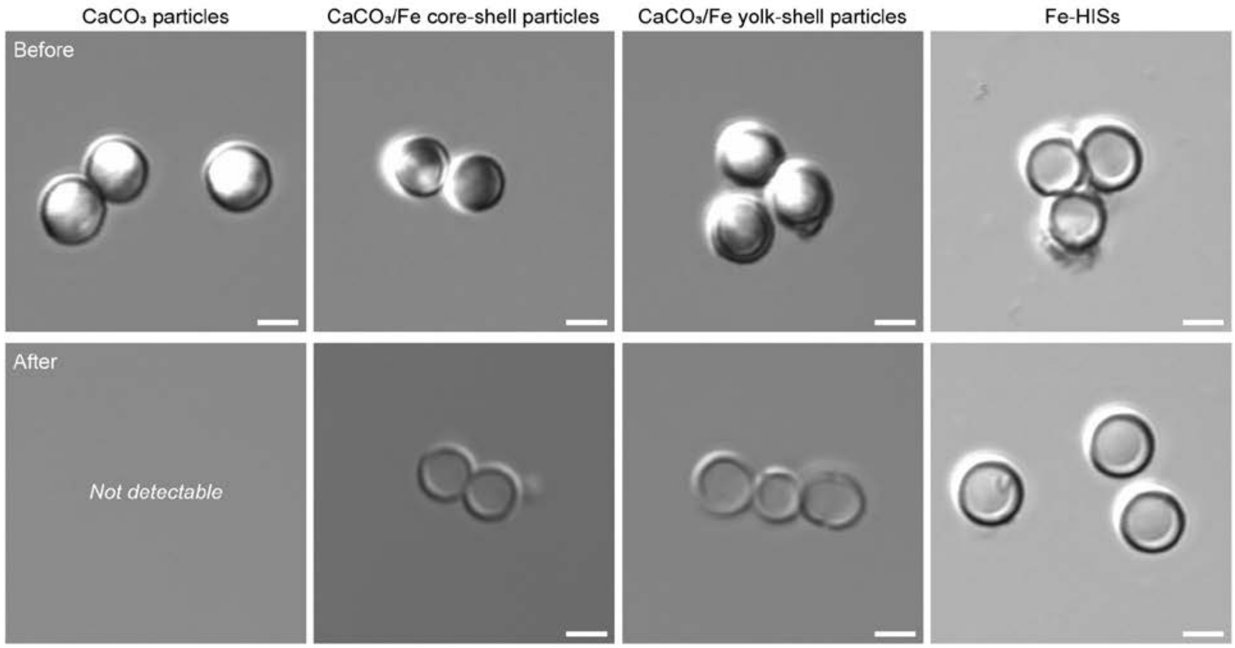

Supplementary Fig. 11: DIC images of $CaCO_3$ particles, $CaCO_3$/Fe particles, and Fe-HISs before (top row) and after (bottom row) EDTA treatment. Scale bar: 2 µm.

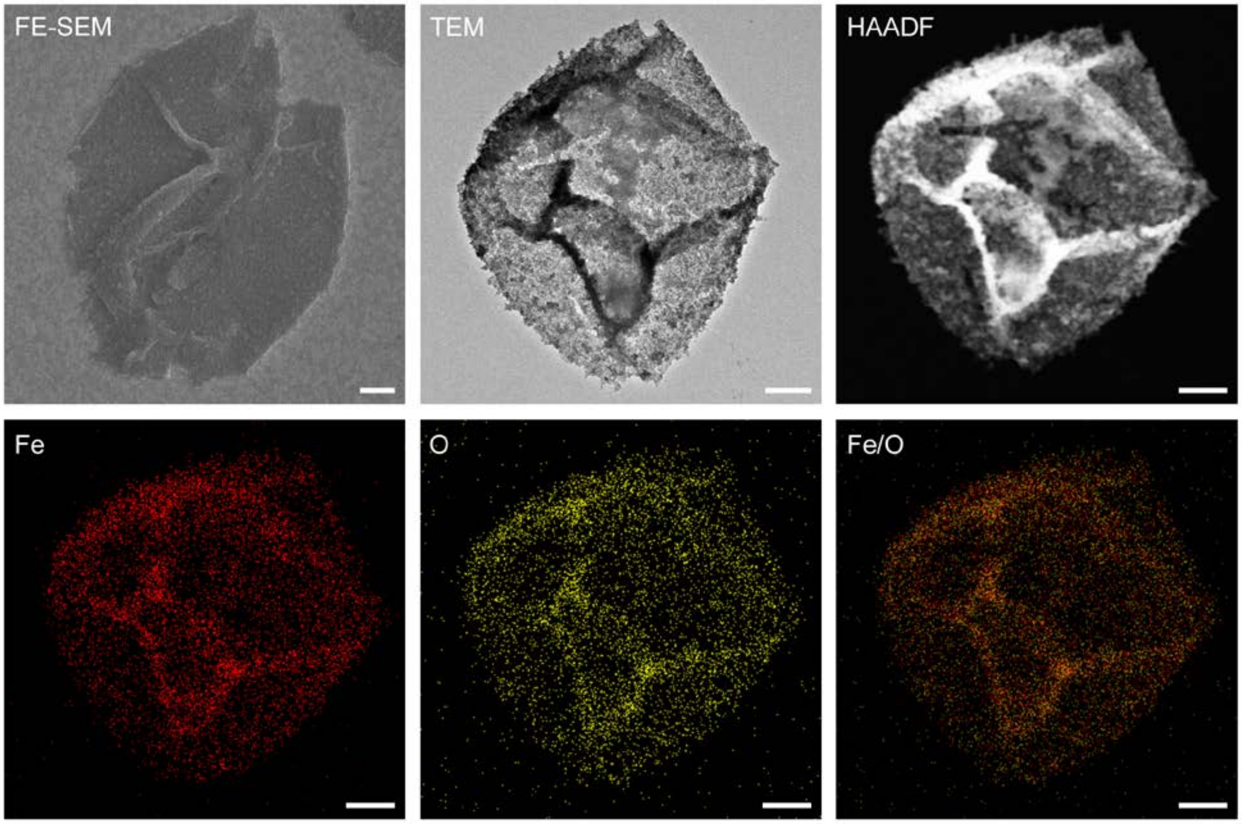

Supplementary Fig. 12: Representative FE-SEM, TEM, and EDX-mapping images of Fe-HISs synthesized from $CaCO_3$/Fe core-shell particles ([Fe^{3+}]: 5mM) by EDTA treatment. The analysis images show folded and wrinkled capsule structures. Scale bar: 0.5 μm.

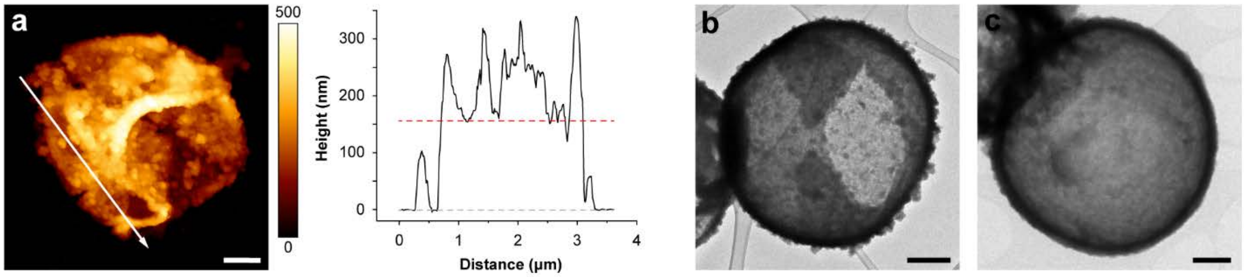

Supplementary Fig. 13: (a) AFM image and height line-profile of an Fe-HIS made with 5-mM Fe^{3+}. Scale bar: 0.5 µm. The red dotted line indicates the height of a bilayer. (b,c) TEM images of Fe-HISs formed with (b) 15- and (c) 17.5-mM Fe^{3+}. Scale bar: 0.5 µm.

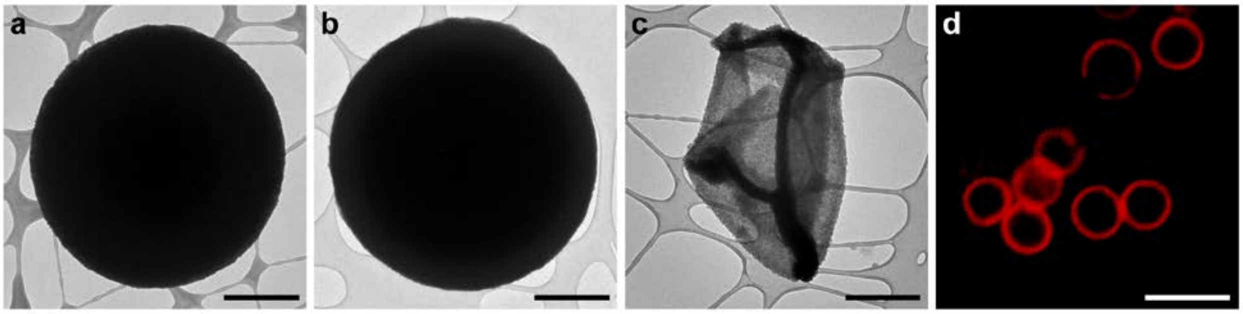

Supplementary Fig. 14: Encapsulation of BSA in the shell of Fe-HISs. (a-c) TEM images of $CaCO_{3[BSA\text{-}rhodamine]}$, $CaCO_3/Fe_{[BSA\text{-}rhodamine]}$, and Fe-$HIS_{[BSA\text{-}rhodamine]}$. Scale bar: 1 µm. (d) CLSM image of Fe-$HIS_{[BSA\text{-}rhodamine]}$. Scale bar: 5 µm.

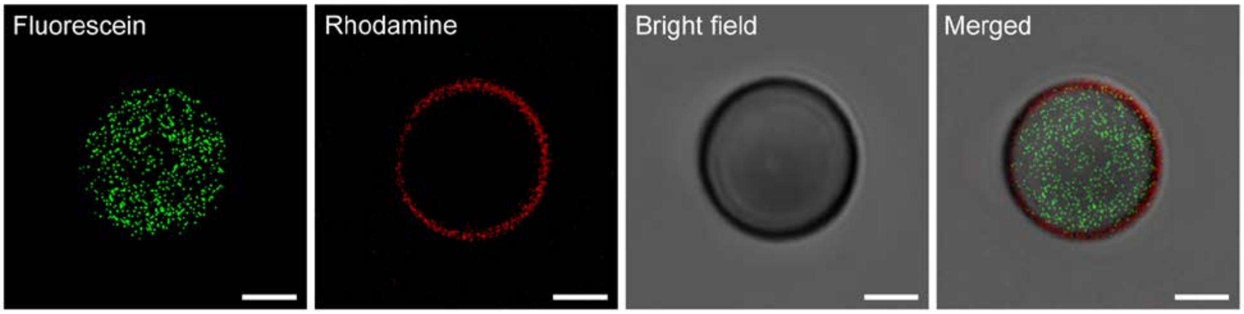

Supplementary Fig. 15: Spatially controlled encapsulation of bioentities in Fe-HISs. CLSM images of an Fe-HIS encapsulating BSA-fluorescein (green) and BSA-rhodamine (red). $CaCO_3$ particles were synthesized in the presence of BSA-fluorescein. Scale bar: 2 μm.

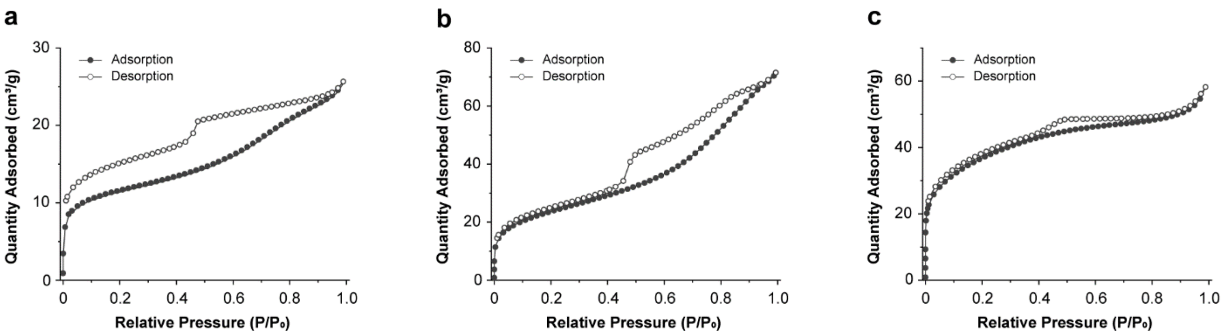

d

	$CaCO_3$	$CaCO_3$/Fe	Fe-HIS
**BET Surface Area (m^2/g)**	42.55	85.46	133.55
**Pore Size (nm)**	3.53	4.93	2.49

Supplementary Fig. 16: Nitrogen adsorption-desorption isotherms of (a) $CaCO_3$, (b) $CaCO_3$/Fe core-shell particles, and (c) Fe-HISs. (d) BET surface areas and pore sizes.

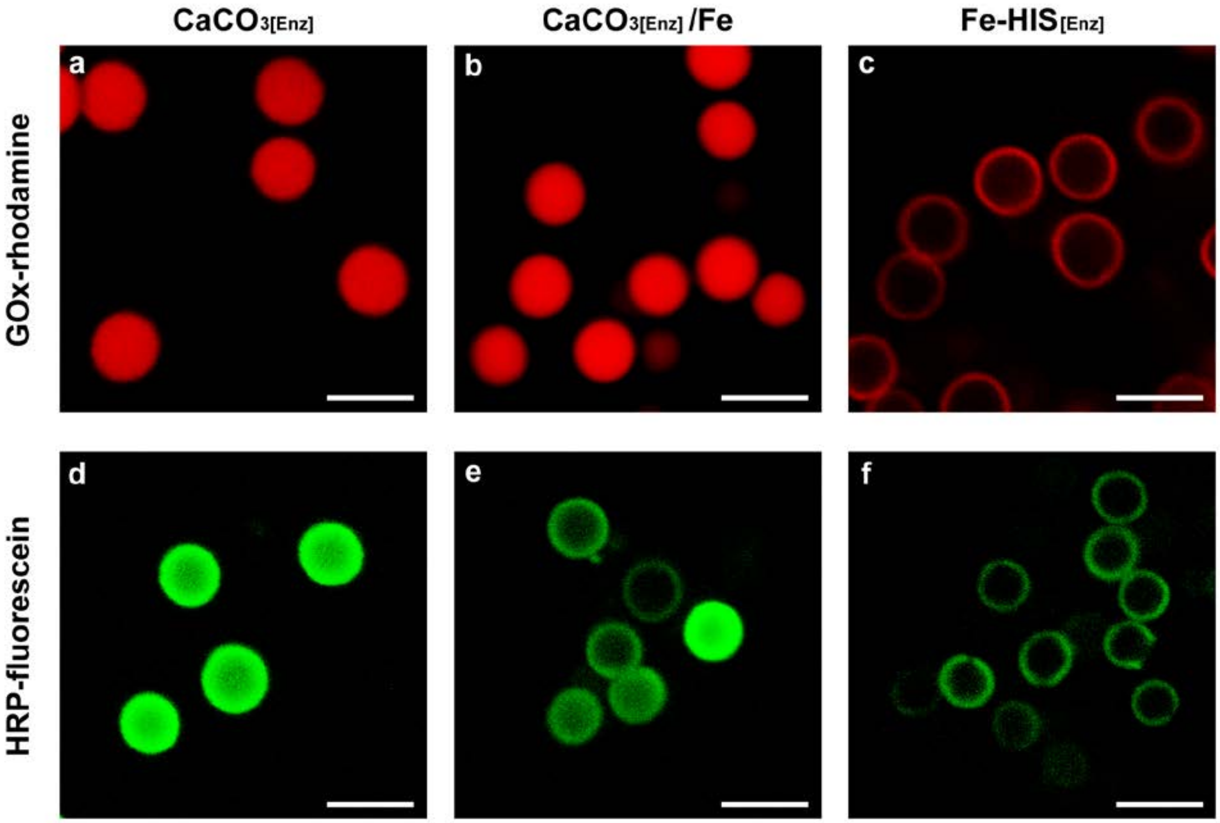

Supplementary Fig. 17: Encapsulation of enzymes in the shell of Fe-HISs. (a-c) CLSM images of $CaCO_{3[GOx\text{-}rhodamine]}$, $CaCO_3/Fe_{[GOx\text{-}rhodamine]}$, and Fe-$HIS_{[GOx\text{-}rhodamine]}$. (d-f) CLSM images of $CaCO_{3[HRP\text{-}fluorescein]}$, $CaCO_3/Fe_{[HRP\text{-}fluorescein]}$, and Fe-$HIS_{[HRP\text{-}fluorescein]}$. Images c and f were taken after 3 h of incubation in DI water. The UV-vis absorbance measurements at 550 nm and 460 nm showed the loading efficiency of 32.9% for GOx and 9.3% for HRP, respectively, in $CaCO_{3[Enz]}$. Scale bar: 5 µm.

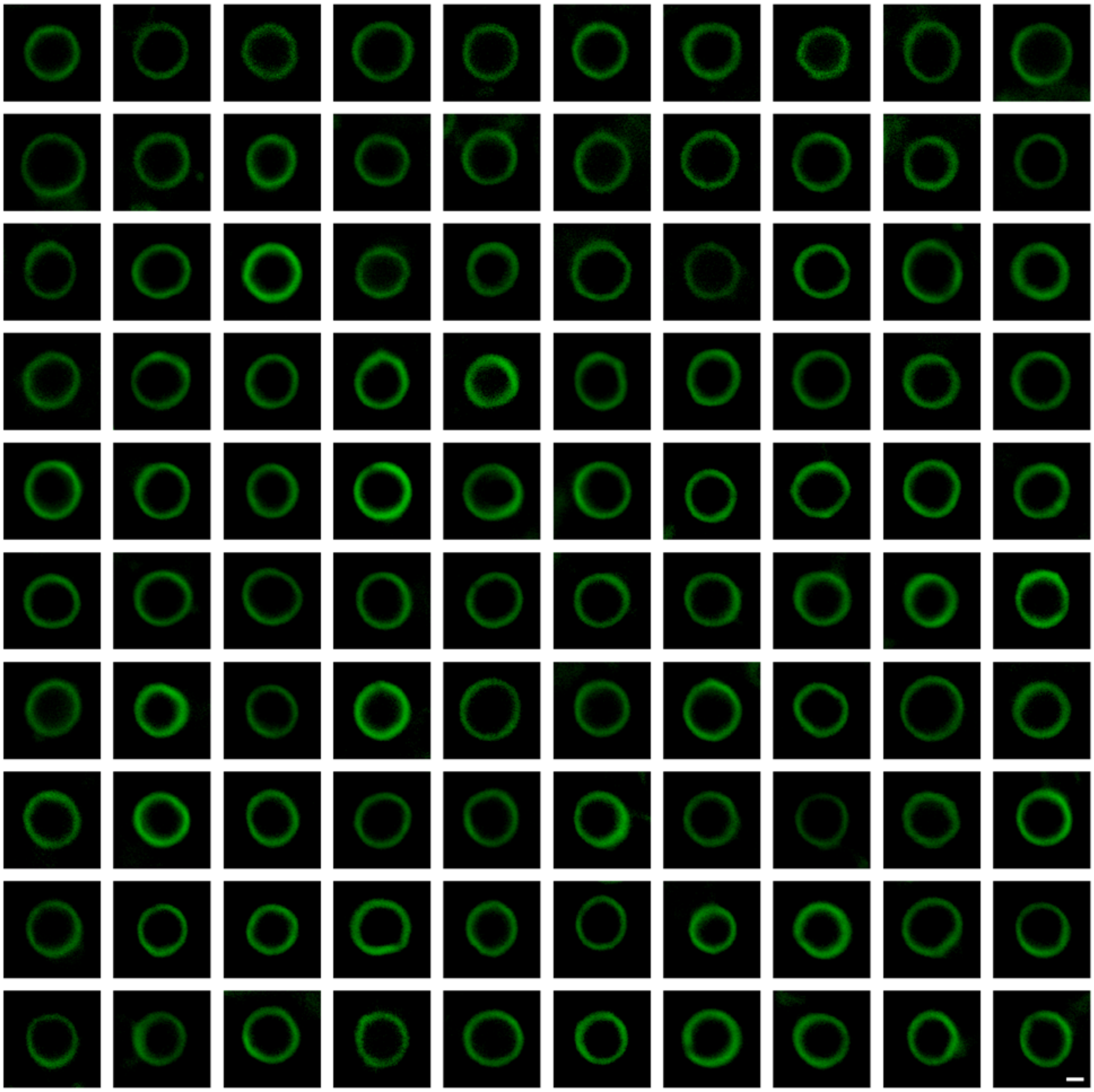

Supplementary Fig. 18: CLSM images of 100 individual Fe-$HIS_{[HRP\text{-}fluorescein]}$ from various batches. Scale bar: 1 µm.

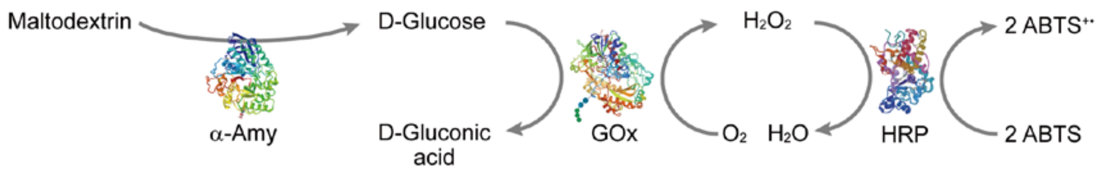

Supplementary Fig. 19: Schematic for a cascade reaction catalyzed by α-Amy, GOx, and HRP.

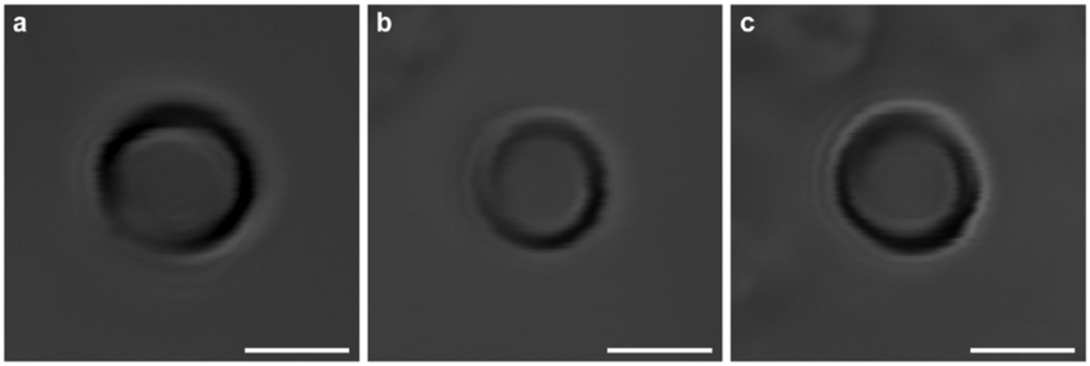

Supplementary Fig. 20: DIC images of (a) Fe-HIS$_{[GOx]}$, (b) Fe-HIS$_{[HRP]}$, and (c) Fe-HIS$_{[GOx/HRP]}$. Scale bars: 2 µm.

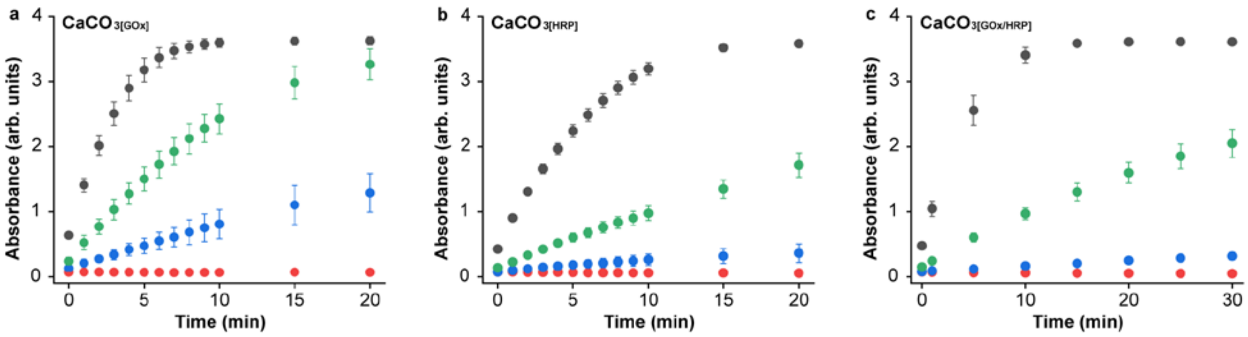

Supplementary Fig. 21: Time-lapse UV-vis absorbance of the ABTS assay solution at 414 nm. Catalytic activities (a) GOx, (b) HRP, and (c) GOx/HRP along with the structural evolution. The reaction rates (unit: abs. min^{-1}) were calculated based on the slopes during the initial 5 min for each sample. Gray circle: free enzymes; green circle: Fe-$HIS_{[Enz]}$; blue circle: core-shell $CaCO_3$/$Fe_{[Enz]}$; red circle: $CaCO_{3[Enz]}$. Data are represented as mean ± SD.

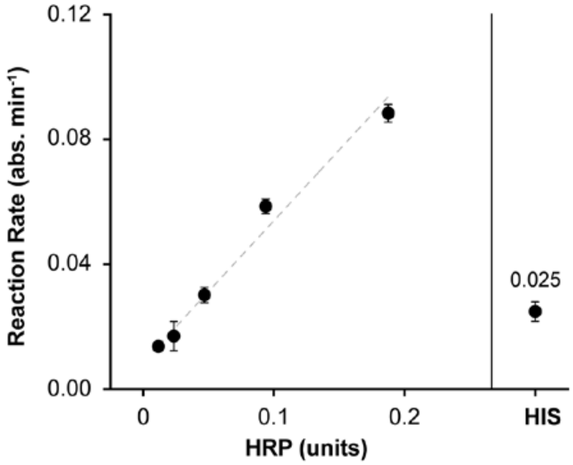

Supplementary Fig. 22: Peroxidase-mimicking catalytic activity of Fe-HISs compared with HRP based on the ABTS assay. Data are represented as mean ± SD.

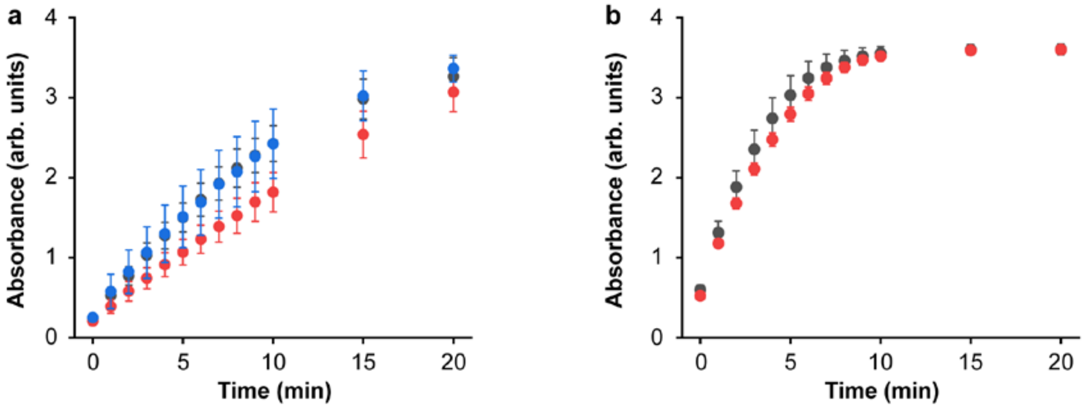

Supplementary Fig. 23: Effects of PSS, Fe^{3+} ions, and EDTA on the activity of GOx within the shell of Fe-HISs. (a) Time-lapse UV-vis absorbance at 414 nm of the ABTS assay solution containing Fe-$HIS_{[GOx]}$ after (blue) EDTA and (red) Fe^{3+} treatment; (gray) intact Fe-$HIS_{[GOx]}$ as a control. (b) Time-lapse UV-vis absorbance at 414 nm of the ABTS assay solution containing free GOx and HRP (gray) without and (red) with PSS. Data are represented as mean ± SD.

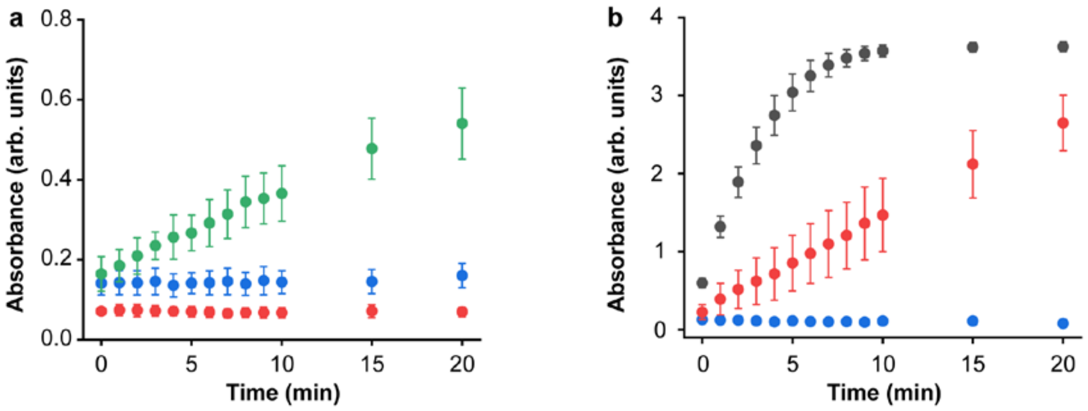

Supplementary Fig. 24: (a) Time-lapse UV-vis absorbance of Ru-$HIS_{[GOx]}$ in the ABTS solution at 414 nm. Green circle: Ru-$HIS_{[GOx]}$; blue circle: core-shell $CaCO_3$/$Ru_{[GOx]}$; red circle: $CaCO_{3[GOx]}$. (b) Time-lapse UV-vis absorbance of Ru-HISs in the ABTS solution at 414 nm. Gray circle: free enzymes; red circle: Ru-HIS with GOx; blue circle: Ru-HIS with HRP. Data are represented as mean ± SD.

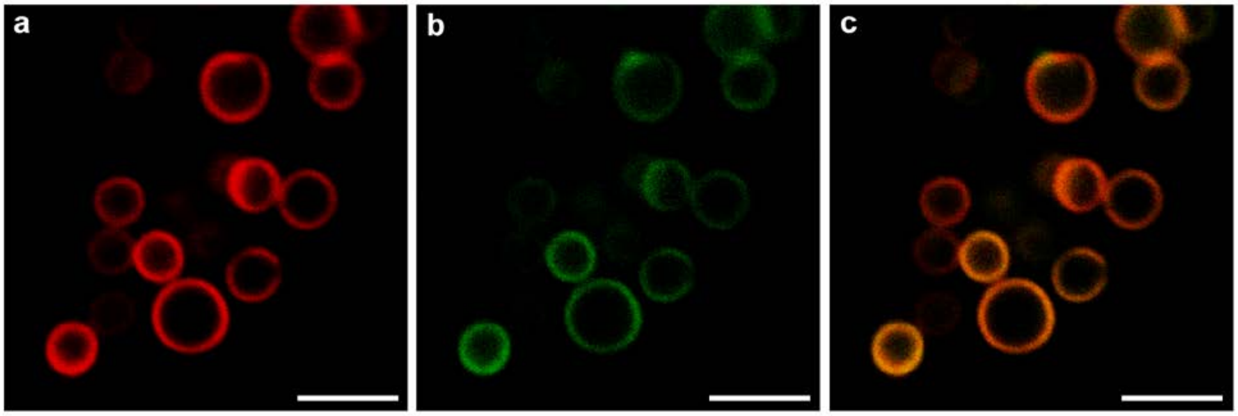

Supplementary Fig. 25: CLSM images of Fe-HIS$_{[GOx\text{-}rhodamine/HRP\text{-}fluorescein]}$. Scale bar: 5 µm.

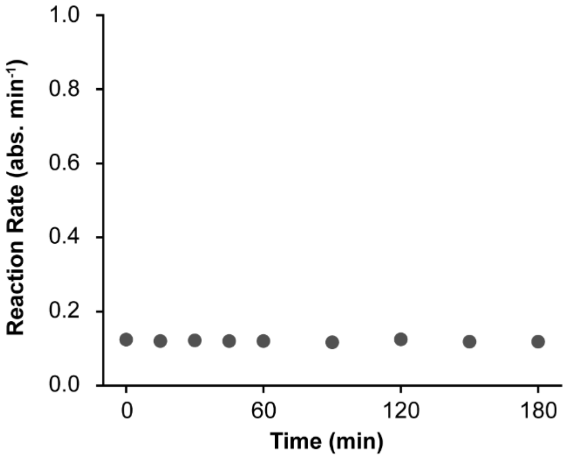

Supplementary Fig. 26: Time-lapse UV-vis absorbance at 414 nm of the ABTS assay solution containing Fe-HIS$_{[GOx/HRP]}$ with maltodextrin. Data are represented as mean ± SD.

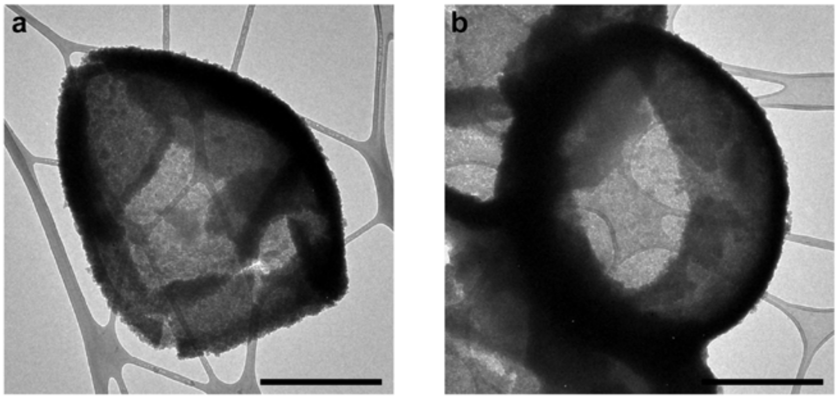

Supplementary Fig. 27: TEM images of Fe-HIS$_{[GOx/HRP]}$ (a) before and (b) after five recycling experiments. Scale bar: 1 µm.
